# The long and short of it

**DOI:** 10.7554/eLife.70757

**Published:** 2021-07-02

**Authors:** Lori A Passmore, Terence TL Tang

**Affiliations:** 1 MRC Laboratory of Molecular BiologyCambridgeUnited Kingdom

**Keywords:** regulation of translation, regulation of mRNA stability, translation, poly(A) tail, PABPC1, *Xenopus oocytes*, Human, Mouse, *Xenopus*

## Abstract

Longer poly(A) tails improve translation in early development, but not in mature cells that have higher levels of the protein PABPC.

**Related research article** Xiang K, Bartel DP. 2021. The molecular basis of coupling between poly(A)-tail length and translational efficiency. *eLife* **10**:e66493. doi: 10.7554/eLife.66493

The genetic code stored in DNA provides cells with the blueprint they need to make proteins. The relevant gene is first transcribed into an intermediate molecule called mRNA (short for messenger RNA), which in turn gets translated by large cellular machines called ribosomes into the string of amino acids that make up a protein. At the end of each mRNA molecule is a poly(A) tail comprised of tens to hundreds of nucleotides called adenosines. The length of these tails can vary greatly between different mRNAs, and longer poly(A) tails are thought to improve translation and increase mRNA stability (Sachs, 1990).

Poly(A) tails stimulate the process of translation through a protein called PABPC (short for Poly(A)-Binding Protein, Cytoplasmic), which binds to the tail (Kühn and Wahle, 2004). This led to the assumption that mRNA molecules with longer tails are translated more efficiently (meaning they produce more proteins) because they can be bound by more copies of PABPC.

However, decades of research on the poly(A) tail have led to inconsistent results: in some experiments, longer poly(A) tails improve translation as hypothesized, while in others they have no effect (Beilharz and Preiss, 2007; Legnini et al., 2019; Lim et al., 2016; Lima et al., 2017; Morgan et al., 2017; Nudel et al., 1976; Subtelny et al., 2014). The contentious role of PABPC in translation efficiency has raised fundamental questions: exactly how important are the poly(A) tail and PABPC in translation, and what are their relative roles in different types of cell? Now, in eLife, David P Bartel and Kehui Xiang from the Whitehead Institute for Biomedical Research and Massachusetts Institute of Technology report new findings that help to answer these questions (Xiang and Bartel, 2021).

To investigate the role PABPC and poly(A) tail length play in translation efficiency, Xiang and Bartel manipulated PABPC levels in *Xenopus* oocytes and in post-embryonic mammalian cells. They then measured the length of the poly(A) tails and compared this to the number of ribosomes occupying each mRNA molecule, which acts as a readout for translation efficiency.

Xiang and Bartel discovered that poly(A) tail length is only linked to translation efficiency when certain criteria are met (Figure 1). They found that when PABPC concentrations were increased in oocytes, mRNAs with shorter poly(A) tails would bind to more ribosomes and show improved translation, whereas those with longer tails were unaffected. Therefore, the first criterion is that PABPC concentrations must be limiting for the protein to preferentially bind to mRNAs with longer poly(A) tails and enhance their translation. Second, for this coupling to occur, mRNA molecules not bound by PABPC must be protected from degradation. Finally, the higher number of PABPC proteins connected to the longer tails must help ribosomes load onto the mRNA molecule more efficiently to increase the amount of protein each mRNA can produce.

**Figure 1.**
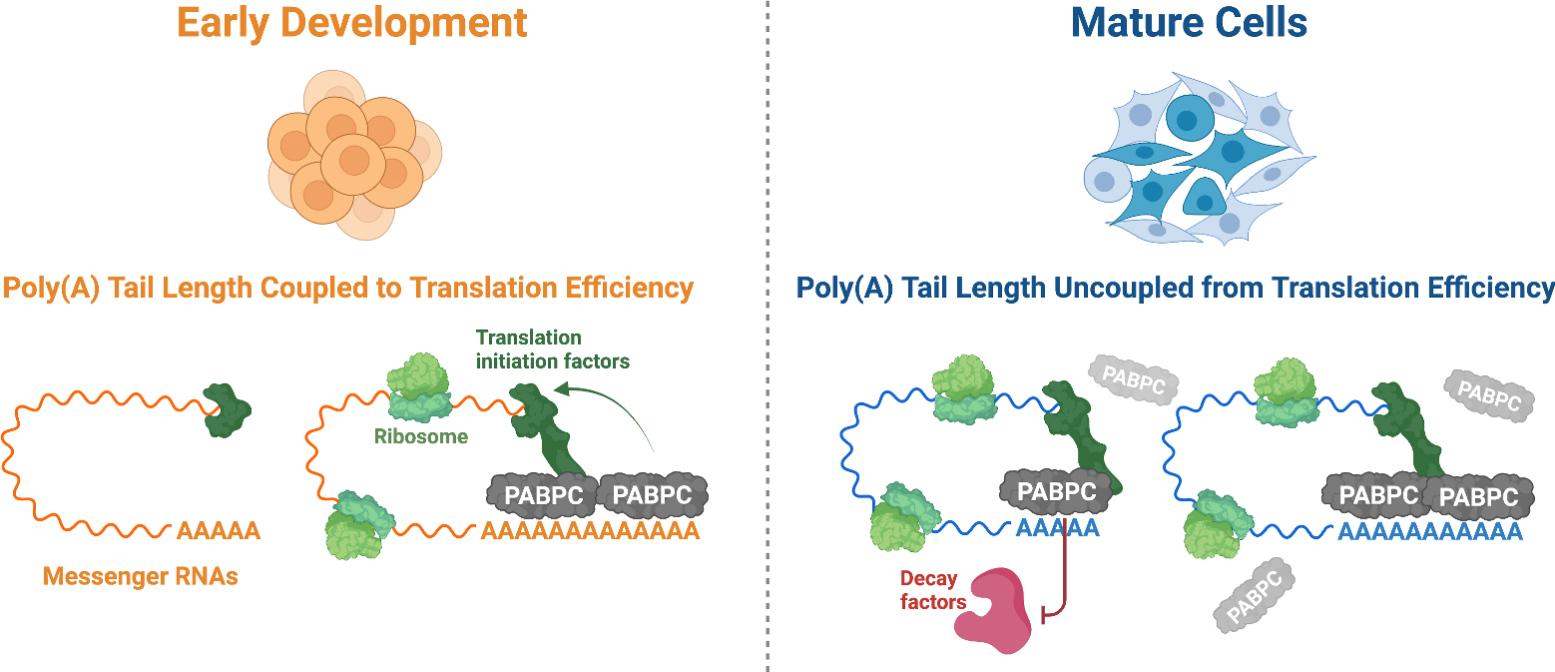
The role PABPC and poly(A) tail length play in translation efficiency changes during development. During early development (left), the amount of PABPC (grey) is limiting. As a result, most of the PABPC available binds to messenger RNAs (mRNAs; orange) with longer poly(A) tails, which helps ribosomes (light green) to translate these molecules more efficiently via improved recruitment of translation initiation factors (dark green). In mature cells (right), the concentration of PABPC is greatly increased, allowing it to bind indiscriminately to all poly(A) tails (blue). As other factors are present to regulate translation efficiency, the importance of PABPC in improving translation is reduced. Instead, PABPC protects mRNAs from degradation, particularly those with shorter poly(A) tails, by outcompeting decay factors (red) for binding sites on the poly(A) tail.

Image credit: Terence Tang, figure created using biorender.com.

In oocytes and early embryos, all three of these conditions are met. This suggests that, during the early stages of development, coupling between poly(A) tail length and translation efficiency plays a primary role in gene expression. However, the relative amount of PABPC protein greatly increases as development progresses, and it eventually occupies every accessible poly(A) tail. Since every mRNA with a poly(A) tail is bound to at least one PABPC, translation no longer depends on the length of the tail, and it is likely that additional factors determine translation efficiency. In this situation, PABPC takes on a new role and instead protects mRNA molecules from destruction, particularly those with shorter poly(A) tails (Figure 1). Its role in stabilising mRNAs could be significant as many ‘housekeeping’ genes, whose expression is required for normal cellular function, tend to have shorter poly(A) tails (Lima et al., 2017).

With exhaustive experiments in different cell types and cellular conditions, Xiang and Bartel firmly establish that the roles of PABPC and poly(A) are highly context-dependent. These results reconcile many years of controversy, by demonstrating that PABPC and the poly(A) tail play different roles in different situations: during the early stages of development, poly(A) tail length and translation efficiency are coupled together by PABPC, while later in development, this relationship is lost and PABPC instead promotes mRNA stability.

Future studies can build upon this foundation by studying how mRNAs with shorter poly(A) tails are stabilised during early development, or which additional factors regulate translation efficiency in mature cells. Furthermore, it remains a mystery how the transition between early and late development is sensed and regulated, leading to a dramatic increase in PABPC levels. Finally, this study raises the interesting possibility that certain cell types, organisms, or cellular conditions exploit the dynamic relationship between PABPC abundance and poly(A) tail length to fine tune gene expression.
